# Differences of Cytotoxicity of Orthodontic Bands Assessed by Survival Tests in *Saccharomyces cerevisiae*

**DOI:** 10.1155/2014/143283

**Published:** 2014-01-06

**Authors:** Tatiana Siqueira Gonçalves, Luciane Macedo de Menezes, Luciele Gonzaga Ribeiro, Catieli Gobetti Lindholz, Renata Medina-Silva

**Affiliations:** ^1^Department of Orthodontics, Dentistry Faculty, Pontifícia Universidade Católica do Rio Grande do Sul, Avenida Ipiranga 6681, Building 6, Room 209, 90619-900 Porto Alegre, RS, Brazil; ^2^Immunology and Microbiology Laboratory, Biosciences Faculty, Pontifícia Universidade Católica do Rio Grande do Sul, Avenida Ipiranga 6681, Building 12, Lab 12D, 90619-900 Porto Alegre, RS, Brazil

## Abstract

The aim of this study was to evaluate the cytotoxicity induced by orthodontic bands through survival tests on *Saccharomyces cerevisiae,* a microorganism that presents several genetic and biochemical characteristics similar to human cells. Three groups of bands were evaluated: silver soldered (SSB), laser soldered (LSB), and bands without any solder (WSB). Yeast cells were directly exposed to the bands and indirectly, when a previous elution of the metals in artificial saliva was performed. The negative control was composed of yeast cells or artificial saliva not exposed to any kind of metal. In the direct exposure experiments, all tested groups of bands induced a slight reduction in yeast viability compared to the control. This effect was more intense for the SSB, although not statistically significant. For the indirect exposure experiments, the SSB induced a statistically significant decrease in cell viability compared to the LSB. There were no significant differences between the survival rates of the negative control and the LSB group in both direct and saliva tests. SSBs were cytotoxic, whilst LSBs were not, confirming that laser soldering may be a more biocompatible alternative for use in connecting wires to orthodontic appliances.

## 1. Introduction

Biocompatibility refers to the ability of a biomaterial to perform its desired function with respect to a medical therapy, without eliciting any undesirable local or systemic effects in the recipient or beneficiary of that therapy but generating the most appropriate beneficial cellular or tissue response in that specific situation and optimizing the clinically relevant performance of that therapy [1]. Corrosion is the main concern when biocompatibility of orthodontic metallic materials is evaluated. The release of several metallic ions [2] may lead to hypersensitivity and allergic reactions, either locally as well as systemically [3].

In daily practice, it is usual to use orthodontic bands during interceptive and corrective treatments. The bands are generally made of stainless steel and are composed of nickel, iron and chromium, and it is considered a biocompatible alloy [4, 5]. However, in several clinical situations, it is necessary to connect orthodontic wires to the bands, especially when auxiliary appliances, such as lingual arches and maxillary expanders, are made. To connect the support wires to the appliances, silver solder is the alloy of choice, due to its proven effectiveness, low cost, and ease of use. However, the silver solder alloy contains silver, copper, and zinc. These ions present a major tendency to be released to the buccal cavity [6] and they may have cytotoxic effects, resulting in decrease of cell viability [5]. Cadmium used to be added to the composition of silver solder alloys some decades ago [7] and, due to the process of zinc obtaining from the ores, cadmium may appear as a zinc contaminant [8]. It is important to remember that cadmium exposure is responsible for hepatic, renal, and myocardial damage characterized by increased creatinine, total and direct bilirubin concentrations and increased ALT and lactate dehydrogenase (LDH) activities [9]. Besides this, cadmium has been considered a mutagen and may be related to the occurrence of cancer [10–12].

An alternative to soldering with silver solder can be the laser welding. In this method, the use of a third metal or alloy, such as the silver solder, can be avoided, as the stainless steel bands and orthodontic wires can be directly connected. With laser soldering, the energy generated promotes real fusion of the metals joined. It may be less susceptible to corrosion and consequently more biocompatible.

Nowadays, several *in vitro* cell culture tests can be used in order to assess the cytotoxicity of dental materials. Among these tests, some yield similar results, whereas some others reveal diverse or even opposing findings [5, 13–17]. The yeast *Saccharomyces cerevisiae* [18] can be used as a model organism for the *in vitro* cytotoxicity evaluation of several harmful agents as well [19–24], offering advantages once they are easy and inexpensive to manipulate. They can provide a large amount of quantitative data from well-controlled experiments with short-time results being phylogenetically very closely related to animals [25]. Biochemical and genetic similarities [26, 27] justify the use of yeast models to address a scientific question of clinical interest [18, 28–33]. However, few dental studies have used this microorganism for this purpose [34, 35], and only one was dedicated to efficiently evaluate orthodontic materials [4].

Taking into consideration the fact that silver solder may present cytotoxic effects, that laser soldering is still an emerging technique in orthodontics and has been scarcely evaluated and, mainly, considering the large scale use of orthodontic bands with silver soldered joints in orthodontic auxiliary appliance and the lack of studies evaluating the cytotoxicity of orthodontic bands, the aim of this study was to evaluate the induction of cytotoxicity by orthodontic bands with or without laser or silver solder using a wild-type *S. cerevisiae *strain as a model organism.

## 2. Materials and Methods 

**Figure 1:**
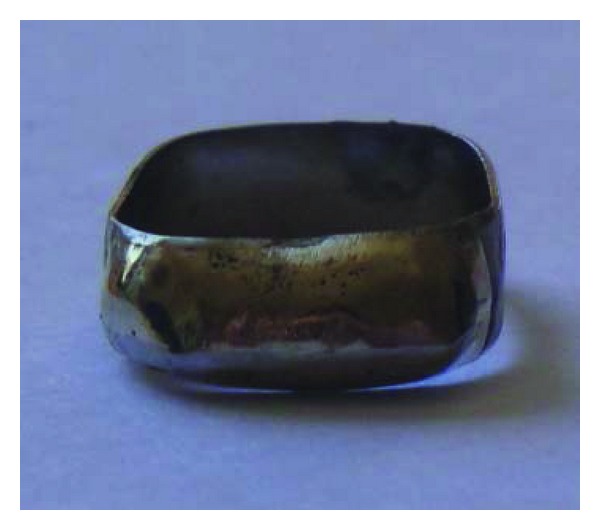
Silver soldered band (SSB).

**Figure 2:**
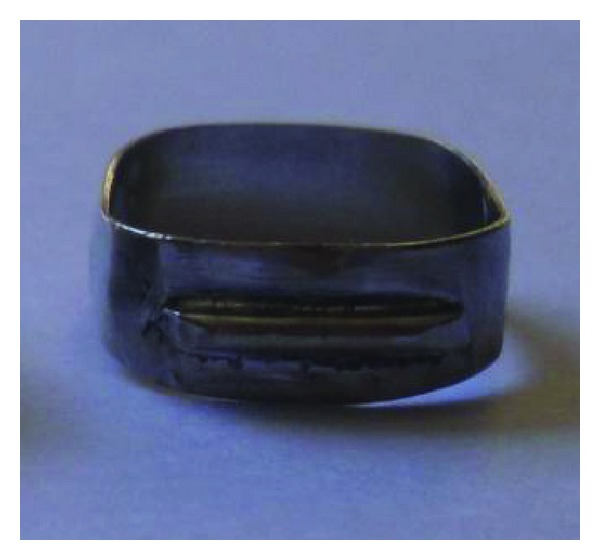
Laser soldered band (LSB).

**Figure 3:**
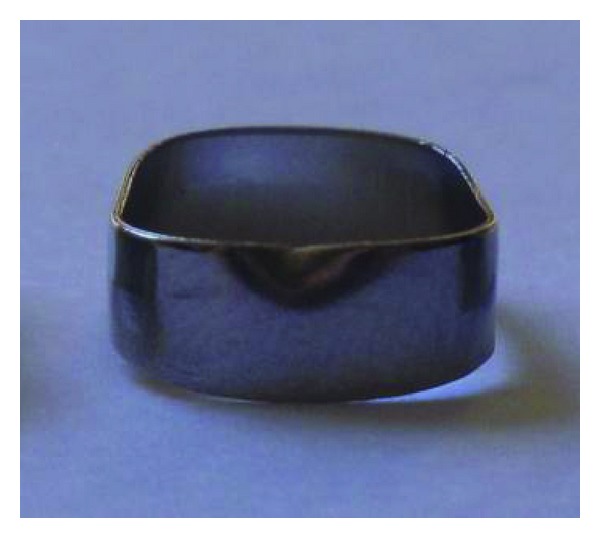
Band without any solder (as received—WSB).

This study was approved by the ethics Committee from Pontifícia Universidade Católica do Rio Grande do Sul (Porto Alegre, Brazil). Stainless steel metallic orthodontic bands (Universal bands for upper molars Morelli, Sorocaba/SP, Brazil) were evaluated. The bands, according to the manufacturer's information, are composed of Cr: 17–20%; Ni: 8–10%; Mo: max. 0,60%; and Fe. Three groups were formed: silver soldered bands (SSB—Figure 1), laser soldered bands (LSB—Figure 2), and bands without any kind of solder (WSB—Figure 3). For the silver solder group, in each band, a segment of stainless steel 1.0 mm wire (Cr: 17–20%; Ni: 8–10%; Mo: max. 0,60%; and Fe) was soldered using silver solder alloy (Ag 55–57%, Cu 21–23%, Zn 15−19%, and Sn 4–6%) and solder flux (Morelli, Sorocaba/SP, Brazil) heated by a butane micro-torch (GB 2001, Blazer, Farmingdale, NY, USA). For the laser soldered group, the same 1.0 mm stainless steel orthodontic wire was soldered to the band using *laser *Nd: Yag (250 V, 12 ms; Dentaurum, DL 2002-S, Germany). The third group was composed of bands without any solder and was evaluated as received.

### 2.1. *S. cerevisiae* Strain, Media, and Cultures

The *S. cerevisiae *strain used in this work was the wild-type strain FF18733. To cultivate this strain, YPD medium (1% yeast extract, 2% peptone, and 2% glucose) was used, either in broth or solid (with agar at 2%) form. In all survival experiments, *S. cerevisiae* precultures were prepared in 10 mL YPD broth and grown overnight to exponential phase (~10^−7^ cells/mL) at 30°C.

### 2.2. Survival Experiments for Cytotoxicity Analysis

The cytotoxicity analysis was performed as already described [4] via two types of survival experiments: (1) direct exposure of *S. cerevisiae* cells to the bands and (2) previous elution of the bands in artificial commercial saliva (Salivan, Apsen Farmacêutica SA, Brazil), followed by exposure of *S. cerevisiae* cells to the artificial saliva containing the metals' elutes. The negative control in the direct exposure was composed of yeast cells that were not exposed to any kind of metal. In the saliva exposure test, the artificial saliva was the negative control. The experiments were performed in triplicate.

*Direct Exposure Experiments.* New inocula were made, each one containing one band either with silver solder (SSB), laser solder (LSB), or without any solder (WSB) and were incubated at 30°C to exponential phase (~10^−7^ cells/mL). Aliquots from each culture were diluted in 0.9% sterile saline solution and 5 *μ*L drops from each dilution (from 10^−2^ to 10^−5^) were plated on YPD agar and incubated at 30°C for two days for the emergence of small colonies, which allowed an initial qualitative approach. For the final quantitative analyses, 100 *μ*L of the final dilutions were plated on YPD agar (two plates for each dilution) for colony counting and CFU/mL estimative after two days at 30°C.

*Saliva Exposure Experiments*. Each band was immersed in 500 *μ*L of artificial saliva for 7 days. A total of 500 *μ*L of the preinoculum was used for each treatment, which was centrifuged (2 min at 2000 g) and resuspended at 100% with the saliva preexposed to the different bands. The cells were then treated for 60 minutes, diluted, and plated in YPD agar as described above, for both qualitative and quantitative analyses. A negative control was performed with the artificial saliva not exposed to any kind of metal and the tests were performed in triplicate.

### 2.3. Data Analyses

The mean and standard deviation of the colony forming units per mL (CFU/mL) counts from three independent repeats of each treatment were compared to their specific controls to verify the occurrence of significant survival differences. If there was at least one log of difference in terms of CFU/mL in treatments in relation to controls, it was assumed a significant difference, which was an indication of cellular toxicity in *S. cerevisiae.*

## 3. Results

**Figure 4:**
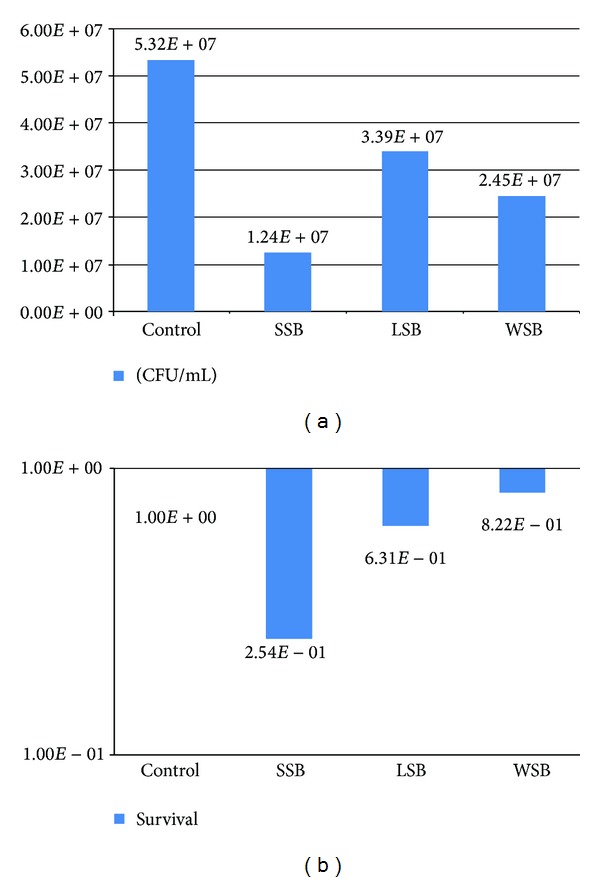
(a) Mean values of cell viability (CFU/mL) from three direct exposure experiments with *S. cerevisiae *strain FF18733 performed with bands bearing silver solder (SSB), laser solder (LSB), or without solder (WSB) in YPD agar. (b) Mean values of yeast survival from three direct exposure experiments with *S. cerevisiae *strain FF18733 performed with bands bearing silver solder (SSB), laser solder (LSB), or without solder (WSB) in YPD agar.

**Figure 5:**
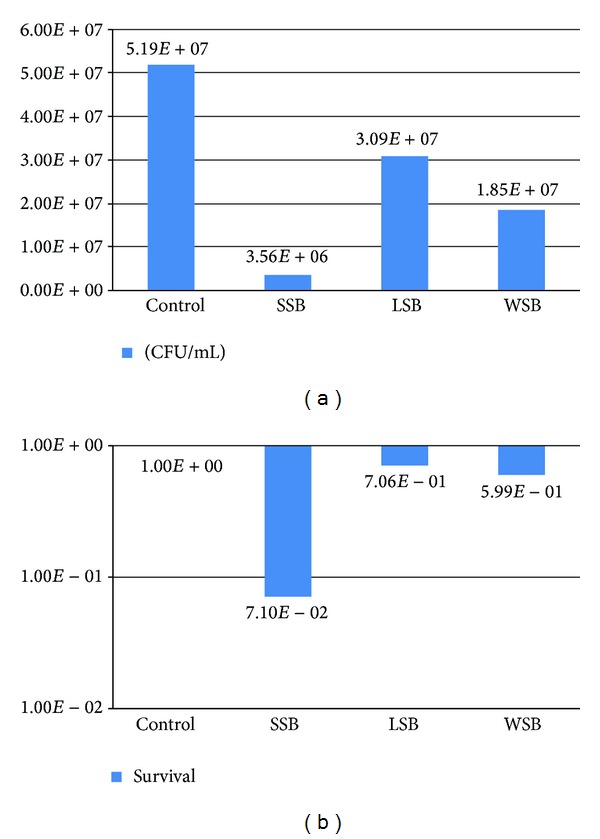
(a) Mean values of cell viability (CFU/mL) from three experiments with saliva elutes in *S. cerevisiae *strain FF18733 performed with bands bearing silver solder bands (SSB), laser solder (LSB), or without solder (WSB) in YPD agar. (b) Mean values of yeast survival from three experiments with saliva elutes in *S. cerevisiae *strain FF18733 performed with bands bearing silver solder (SSB), laser solder (LSB), or without solder (WSB) in YPD agar.

The results from survival experiments are shown in Figures 4 and 5. Regarding the direct experiments, it is possible to observe that the three groups (SSB, LSB, and WSB) induced a decrease in cell viability of *S. cerevisiae* in terms of CFU/mL compared to the control. This effect was more intense with the SSB group, which can be viewed in terms of viable cells (Figure 4(a)). Nevertheless, there was no significant difference in terms of survival, since it was below one log of difference for all samples, but SSB, the one that bear more metal alloys, achieved the higher value (Figure 4(b)). The experiments of saliva exposure showed that the saliva elutes from the three different groups are also able to induce a decrease in *S. cerevisiae* cell viability (Figure 5(a)). The SSB samples were also those that most induced cytotoxicity and, in this case, with a significant difference in terms of survival compared to the control, which did not occur with the LSB or the WSB samples (Figure 5(b)). It is important to notice that the data shows no significant differences between the survival results from the LSB (as well as from WSB) in relation to controls in both direct and saliva tests. Moreover, in saliva experiments the difference between the SSB and the LSB in terms of survival percent is considered significant. These results indicate an important difference in terms of cytotoxicity induction between these two kinds of orthodontic joints and thus an indication of higher biocompatible properties of LSB compared to the most used worldwide, the SSB.

## 4. Discussion

An important part of the population undergoes orthodontic treatments during their lives. Orthodontic bands, composed of iron, nickel, and chromium, are frequently joined to orthodontic wires for the making of auxiliary appliances and, for this, it is usually employed a filling material such as the silver solder alloy. This alloy contains silver, copper, and zinc and may even contain a little amount of cadmium. These ions, together with nickel and chromium, may illicit several undesirable reactions. Specifically, when these metals are heated, the corrosion process may be increased, leading to the elution of ions to the buccal cavity, with local and systemic effects [2, 4, 5, 15, 16, 36–41]. In recent years, the use of laser solder has increased, especially for implant-based prosthesis and it can be used for orthodontic purposes as well [42]. It is a very interesting alternative to connect thick wires such as those used in auxiliary orthodontic appliances. The main advantage is that the energy generated by the laser produces a real fusion between the metals connected, avoiding the need of an additional filling material such as the silver alloy. Consequently, the variety of metallic ions is reduced and the corrosion process is lower. However, its cost is still high since there is the need of a very specific equipment to perform it [42].

The experimental model *S. cerevisiae* has been widely used in biomedical research studies, with very diverse objectives and applications, from cellular biology involved in genetic and neurological diseases [43] to toxicological surveys [44]. The broad applicability of this yeast species as a model organism is based on its easy cell cycle control, great facility of biochemical and genetic manipulation, short time, and inexpensive reproducible experiments [45] as well as biochemical and genetic similarity to animal cells [26, 27]. These *S. cerevisiae* properties' enables the achievement of results compatible with other experimental models such as cultured animal cell, such as fibroblasts, osteoblasts, and keratinocytes [15, 39, 40]. Moreover, it proved to be effective to evaluate the cytotoxicity induction of several orthodontic materials [4].

Based on the wide advantages of the biological model described above, the experiments were conducted using both direct exposure of *S. cerevisiae* cells to the bands and also the exposure of these yeast cells to artificial saliva containing the bands' elutes. This second group of experiments was performed in order to simulate the oral cavity chemistry and its effects over the materials tested.

Auxiliary orthodontic appliances with orthodontic bands may stay in the patient's mouth for a long period of time. For patients subject to maxillary expansion and protraction, at least 13 months with the appliance are necessary. When lingual arches are used as space maintainers, it may be used from as early as six years, until the end of the orthodontic treatment, what may occur only at 13-14 years old. For this reason, it is important to investigate cellular effects of the orthodontic bands, as well as their joins, mainly due to the lack of information in the literature concerning specifically this material. The current available reports evaluated mainly orthodontic wires with soldered connections [39–41].

In the SSB group, the bands tested contained silver flux and suffered the effects of the heat and the high temperatures achieved which are necessary to melt the silver alloy. The objective was to reproduce what actually occurs when auxiliary appliances are made, instead of testing the cytotoxic effects of silver solder alloy alone [4, 15]. Lower cell viability was observed in both experiments and with significant differences (higher than 1 log—Figure 5(b)) in relation to the control in the experiments of exposure to saliva elutes, in accordance with a previous study [4]. Possibly, when the bands were in contact with the artificial saliva, corrosion occurred, leading to the elution of toxic ions. Specifically, nickel, a major component of stainless steel bands, may be easily released [46] leading to toxic effects [38, 47, 48]. The components of silver solder alloy may release toxic ions as well [49]. It has been stated that one of the mechanisms involved in the silver solder toxicity is the occurrence of oxidative stress [4].

Solmi et al. [40] evaluated the reaction of fibroblasts cultured *in vitro *in direct contact with samples of soldered and laser-welded joints from orthodontic lingual arches. Adhesion, morphology, and proliferation of the cells were evaluated under contrast phase light microscopy and scanning electron microscopy and it was concluded that laser-welded joints were superior in terms of biocompatibility. The results of Solmi et al. [40] are in accordance with the findings of the present study; however, the authors evaluated the fibroblast's reaction to the soldered surface only, not considering the whole band. It is important to consider that the oxidation process occurs at the whole surface of the band which is in contact with saliva during clinical use, suffering the effects of the corrosion all over the band, involving not only the silver solder metals but the stainless steel components as well. It seems that evaluating the cell survival after an elution time of the materials in artificial saliva, as performed in the present study, is a good alternative to simulate the effects of a liquid immersion media in corrosion of the bands.

Sestini et al. [39] evaluated orthodontic wires and their effects on osteoblasts, fibroblasts, and keratinocytes through several *in vitro* cytotoxicity tests. They found high cytotoxicity of silver soldered joints, whereas laser soldered joints were considered the only joining process well tolerated by all cell types. Again, the findings of Sestini et al. [39] agree with the findings of the present study; however, similar to Solmi et al. [38], the authors evaluated only the wires and did not consider the joining process that occurs in orthodontic bands, which presents a higher area of soldering. The authors used the wires in direct contact with the cells not considering a previous corrosion process, as reported in the present study for the indirect experiments.

As done by Sestini et al. [39], Vande Vannet et al. [41] also evaluated orthodontic wires but used three-dimensional oral mucosal cell. The authors revealed that silver soldered wires led to higher loss of viability than laser welded and electric welded joints. They also tested stainless steel wires alone, as we did with the WSB, assuring the biocompatibility of stainless steel alone. The same good performance was observed for the laser soldered wires, in accordance with the LSB group in our work with the bands. Vande Vannet et al. [41] also found lower cell viability with the silver soldered wires, however, with no statistical differences when compared to the control and to the other tested groups, such as laser solder and stainless steel alone.

In the present study no significant differences were observed between the results of cell survival from the LSB and those from the control, in both direct and indirect evaluations. This indicates that laser soldering was not cytotoxic to *S. cerevisiae* cells. Additionally, there was a significant difference from the levels of cytotoxicity induced by the SSB group in saliva experiments when compared to the LSB, which confirms laser soldering as an interesting alternative for clinical use in orthodontic bands and for the making of auxiliary appliances that are extensively used in clinical practice.

The present study clearly indicated that silver solder actually presents cytotoxic effects and that laser solder is certainly a more biocompatible option for the connection of wires and for auxiliary appliances. However, more studies are necessary using yeast cells or other experimental models to observe not only the cytotoxic effects of silver solder but also if this material actually increases the occurrence of oxidative stress and if that mechanism may lead to possible genotoxic effects.

## 5. Conclusions

Silver soldered bands were cytotoxic to *S. cerevisiae* cells. There was significant difference between the laser soldering and the silver soldering groups, indicating the use of laser soldering as a more biocompatible alternative for clinical use in orthodontic appliances.
